# Dynamic Changes of Circulating* Mir-155* Expression and the Potential Application as a Non-Invasive Biomarker in Breast Cancer

**DOI:** 10.31557/APJCP.2020.21.2.491

**Published:** 2020

**Authors:** Sumadi Lukman Anwar, Dewi Sahfitri Tanjung, Meutia Srikandi Fitria, Aprilia Indra Kartika, Dwi Nur Indah Sari, Dinna Rakhmina, Tirta Wardana, Indwiani Astuti, Sofia Mubarika Haryana, Teguh Aryandono

**Affiliations:** 1 *Division of Surgical Oncology, Department of Surgery, *; 2 *Graduate Program, *; 7 *Departement of Pharmacology and Therapy,*; 8 *Department of Histology and Cell Biology, Faculty of Medicine, Public Health and Nursing, Universitas Gadjah Mada, Yogyakarta, *; 3 *PT Etana Biotechnologies Indonesia, Jakarta, *; 4 *Medical Laboratory Technology, Health and Nursing Faculty, Universitas Muhammadiyah Semarang, Semarang, *; 5 *Fakultas Ilmu Kesehatan, Universitas Setia Budi, Surakarta, *; 6 *Politeknik Kesehatan Kemenkes Banjarmasin, Banjarmasin, Indonesia. *

**Keywords:** microRNA, miR-155, breast cancer, biomarker

## Abstract

**Background:**

Breast cancer incidence rates have been continuously increasing in majority nations with significant higher portion of cancer-related mortality in low- and middle-income countries. Developing new biomarker is an emerging field in the breast cancer research. Application of a promising minimally invasive biomarker, circulating microRNA, for additional improvement of diagnosis, prognosis, and therapeutic monitoring in breast cancer is not well corroborated.

**Materials and Methods:**

To uncover the potential use of circulating *miR-155* expression as a clinical biomarker in breast cancer, we analyzed 102 breast cancer patients at diagnosis and after treatment as well as 15 healthy women. Total RNA was isolated from patient’s plasma and expression of circulating *miR-155* was measured with quantitative reverse transcription polymerase chain reaction (qRT-PCR). The expression levels of circulating miR-155 were compared according to the effect of treatment, clinicopathological variables, and progression-free survival.

**Results:**

In comparison to the healthy women, expression of circulating miR-155 levels were significantly higher (medians were 18.49±19 and 1.28±0.18, respectively; p<0.0001). The expression levels of *miR-155* were significantly diminished after patients completed surgery and chemotherapy (medians were 18.49±19 at diagnosis and 1.32±0.22 after treatment, respectively; p<0.0001). Patients older than 40 years old expressed higher circulating miR-155 than those younger than 40 years-old (medians were 28.92±22 and 4.19±2.49, respectively; **p<0.0001). **Circulating *miR-155* was significantly higher in patients with tumors larger than 5 cm (44.27±2.6 vs 9.17±6.9, p=0.03). *MiR-155* expression levels were not significantly different according to various tumor grades, subtypes, and clinical stages. Although longer follow-up is required, progression-free survivals of patients with upregulation of circulating *miR-155* were significantly longer (mean survivals were 77 and 65 weeks, Log-rank (Mantel-Cox) test p=0.038).

**Conclusion:**

Expression of circulating *miR-155* expression was significantly elevated in breast cancer patients and was decreased after treatment. Therefore, circulating* miR-155* is potentially applicable as diagnostic therapeutic monitoring marker in breast cancer.

## Introduction

The incidence rates of breast cancer have been increasing almost in all countries to reach more than 2 million cases in 2018 (Bray et al., 2018). In addition, breast cancer has emerged as the most frequent cause of cancer-associated mortality in developing countries and the second after lung cancer in more developed countries (Bray et al., 2018). The implementation of national screening program in developed countries has shifted the diagnosis into more early stages (Youlden et al., 2012). However, breast cancer in developing countries including in Indonesia is mostly still diagnosed in the late stages (Akhsan and Aryandono, 2010; Anwar et al., 2019a). Low cancer awareness and lack of national early detection and screening program (Anwar et al., 2018; Anwar et al., 2018) have been associated with late presentation and delayed diagnosis in low- and middle-income counties (Rivera-Franco and Leon-Rodriguez, 2018). In addition, the high proportion of mortality rates is associated with late stages at diagnosis, relapse, and distant metastasis. 

Given the substantial health burdens of breast cancer, there is an increasing need to develop reliable biomarkers to improve the chance of early detection, to determine patient prognosis, and to predict drug resistance and relapse (Duffy et al., 2018). The new biomarker is expected to detect breast cancer before the disease spreads beyond the breast or the axillary lymph nodes (Duffy et al., 2018) and to accurately detect any relapse or metastasis before the emerging of clinical manifestation. Currently, only Cancer Antigen 15-3 (CA 15-3) has been used as circulating marker to predict relapse in the clinical practice although lacking of sensitivity and specificity (Atoum et al., 2012). To overcome this limitation, new reliable non-invasive biomarker for breast cancer patients is needed.

A new class of small noncoding RNAs, known as microRNAs, is a master regulator of gene expression that can target multiple protein encoding genes (Winter et al., 2009). MicroRNAs inhibit gene expression post transcriptionally through direct interaction causing mRNA degradation or translational inhibition depending on the cross-matching of the seed sequence and the 5’ or 3’ mRNA targets (Winter et al., 2009). Unique patterns of microRNA expression in primary tumor tissue samples have been revealed as a potential biomarker in various cancers including breast cancer (Croce, 2009; Volinia et al., 2012). The deregulation of micoRNA in primary cancer tissues results from defect of microRNA biogenesis and transcription factors (Winter et al., 2009), epigenetic regulation (Anwar et al., 2013), genetic amplification/deletion (Shimizu et al., 2004), and interaction of SNPs at the flanking region (Anwar et al., 2017). Interestingly, microRNAs can be exported from malignant cells into the extracellular fluid and circulating through several modes including micro-vesicles, lipid vesicles, exosomes, and apoptotic bodies (Turchinovich et al., 2012). Because RNAs are easily degraded by endogenous RNase, extracellular microRNAs are viably observed forming a protein complex with ribonucleoprotein or high-density lipoproteins (Roth et al., 2010). As circulating microRNA reflects biological characteristics of the underlying tumor, there is substantial interest to use it as a non-invasive marker in breast cancer. MiR-155 is an important oncogenic microRNA in human cancers including in breast cancer (Khalighfard et al., 2018). Although the upregulation in primary tumor tissues of breast cancer patients has been frequently reported, the deregulation of circulating miR-155 and the application as clinical biomarker have not been completely revealed. In this study, we measured circulating miR-155 in breast cancer patients at diagnosis and after surgery and chemotherapy and correlated with the clinicopathological variables and progression-free survival.

## Materials and Methods

*Patient cohort*

Patients diagnosed with breast cancer in 2014-2015 were recruited to participate in this study according to a protocol accepted by the Ethics Commission of the Faculty of Medicine, Universitas Gadjah Mada, Indonesia (Number: 601/EC/2014). In total, periphery blood samples were collected from breast cancer patients at diagnosis (N=102) and at follow-up after completion of surgery and chemotherapy (N=40) as well as healthy controls from females without a history of tumor (N=15). Diagnosis of invasive breast cancer was confirmed with the histopathology and only patients that were older than 18 years-old and were able to provide inform consent were than recruited. Vulnerable patients and participants including those with dementia, terminally ill, and in clinical emergency were excluded. Estimation of sample size was calculated using effect size 0.5 of Cohen’s approach with α=0.05 and statistical power of 0.9 reveling sample size needed of 49 patients. Clinicopathological variables including, tumor size, lymph node infiltration, status of local and distant metastasis, immunohistochemistry, and demographic data were collected at the time of diagnosis. Patients were followed-up according to the hospital recommendation. Progression-free survival (PFS) was calculated as a period between time at diagnosis to the time of any relapse or local/regional/distant tumor spreading was observed (until December 2017). Baseline clinicopathology characteristics of breast patients participated in this study was summarized in the Table 1.

*RNA isolation from plasma*

After peripheral venous blood was drawn from the study participants and collected at the EDTA-contained tubes, fractionation was performed by centrifugation of 1,500 rpm for 10 minutes at 4^o^C. The plasma was collected from the supernatant and then stored at -80^o^C until analysis. Total RNA was extracted from 200 µL plasma of each study participant using miRCURYTM RNA Isolation Kit-Biofluid (Exiqon, Cat No. 300112) following recommendation from the manufacturer. Briefly, plasma was mixed with Lysis and Protein Precipitation Solution and then centrifugated at 12,000 rpm. The RNA was precipitated by mixing the supernatant with 270 µL isopropanol and was then separated using microRNA Mini Spin Column (Exiqon). After 2 washing steps, the RNA was eluted using 25 µL RNA-se free HPLC water. Internal control of RNA isolation was controlled using spike-in of Sp6. 

*Quantification of miR-155 expression*

Reverse transcription using Universal cDNA Synthesis kit II, 8-64 rxns (Exiqon, Cat No.203301) was performed to produce cDNA from the RNA derived from plasma. Expression levels of circulating miR-155 were quantified using ExiLent SYBR Green master mix (Exiqon, Cat No. 203402) and specific primers for miR-155 (Exiqon, Cat YP00204308), Sp6 (Exiqon, Cat YP00203954), and MIR-16 (Exiqon, YP00205702). 

*Statistical analysis*

The expression levels of miR-155 were calculated from Cq values and were normalized with Cq values of spike-in Sp6 and miR-16. Relative expression was then compared with the ΔΔCq in healthy individuals. Fold expression values were calculated using the 2 ΔΔCq method. Comparison of circulating* miR-155* expression levels between breast cancer patients and healthy women as wells baseline and after treatment were compared using the Mann-Whitney-U test. Upregulation was categorized as mean of circulating *miR-155* expression + 2xSD. Comparison and analysis of survival was performed using Kaplan-Meier plot and Log-rank (Mantel-Cox) test. All statistical tests were performed with 2-sided and a p-value < 0.05 was considered as statistically significant. Statistical analysis was performed using GraphPad Prism (La Jolla CA, USA) and SPSS 17.0 software (IBM, USA).

## Results

*Upregulation of plasma miR-155 in breast cancer patients*

**Figure 1:**
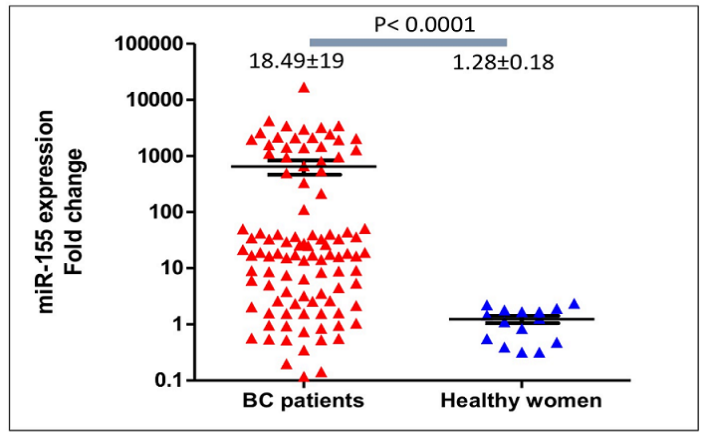
Circulating *miR-155* Expression Levels in Breast Cancer Patients were Frequently Upregulated and were Significantly Higher Compared to Healthy Women. Medians of* miR-155* expression levels were 18.49±19 and 1.28±0.18, respectively; p<0.0001

**Figure 2:**
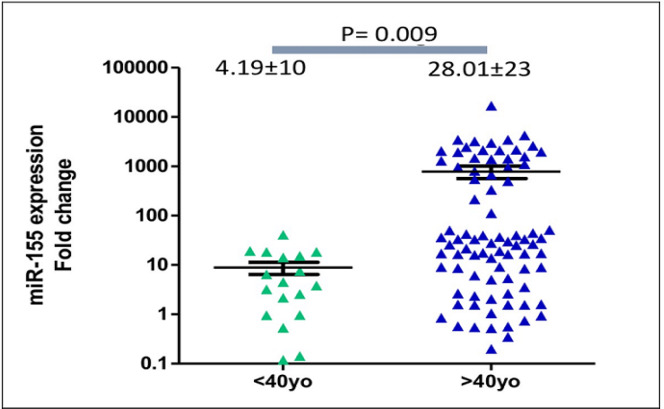
Expression Levels of Circulating *miR-155* were Significantly Higher in Breast Cancer Patients Older than 40 Years-Old (median expression levels were 28.92±22 and 4.19±2.49, respectively; p=0.0009).

**Figure 3:**
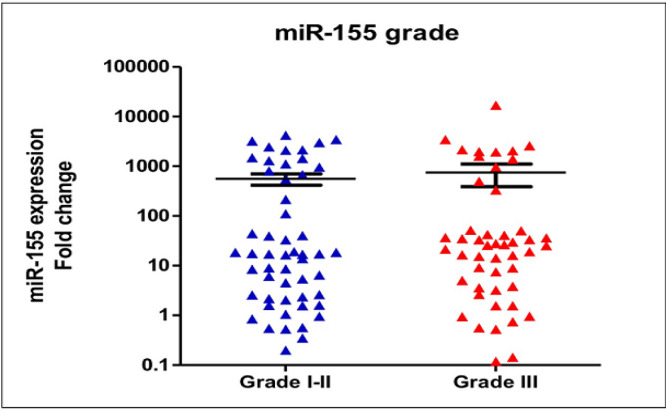
Expression Levels of Circulating miR-155 were not Significantly Different between Good/Moderate Differentiation (Grade I-II) and Poor Differentiation (Grade III) (median expression levels were 17.13±14 and 25.87±35, respectively; p=0.67)

**Figure 4:**
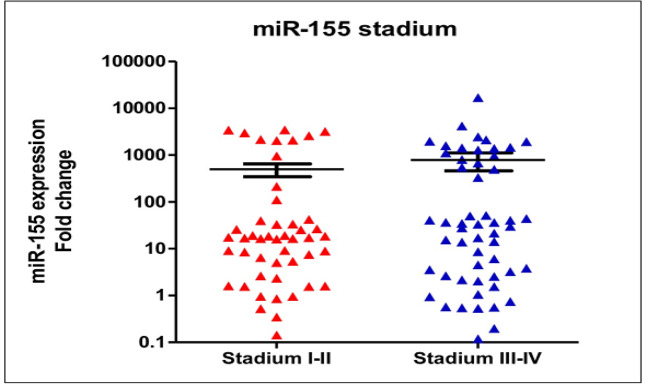
Expression Levels of Circulating *miR-155* were also not Statistically Different between Early Stages (Stadium I-II) and Late Stages (Stadium III-IV) (median expression levels were 17.13±15 and 31.62±13, respectively; p=0.52).

Quantification of circulating miR-155 in breast cancer patients (n=102) revealed a significant upregulation in comparison to the healthy women (n=15) (median expression levels were 18.49±18 and 1.28±0.7, respectively; p<0.0001, see Figure 1). Medians of age of the healthy controls and breast cancer patients were 51±0.7 years-old and 56±0.2 years-old). In breast cancer patients older than 40 years-old, expression levels of circulating miR-155 were significantly higher than those younger than 40 years-old (median expression levels were 28.92±22 and 4.19±2.49, respectively; p<0.0001), Figure 2. In addition, expression of circulating miR-155 was significantly higher in patients younger than 40 in comparison to healthy women (median expression levels were 4.19±2.49 and 1.28±0.7 respectively, p=0.002). According to the histopathological grades, expression levels of circulating miR-155 were not significantly different between good/moderate differentiation (Grade I-II) and poor differentiation (Grade III) (median expression levels were 17.13±14 and 25.87±35, respectively; p=0.67), Figure 3. Expression levels of circulating *miR-155* were also not statistically different between early stages (Stadium I-II) and late stages (Stadium III-IV) (median expression levels were 17.13±15 and 31.62±13, respectively; p=0.52), Figure 4. Circulating miR-155 was significantly higher in patients with tumors larger than 5 cm (44.27±2.6 vs 9.17±6.9, p=0.03) and was not statistically different between triple negative breast cancer (TNBC) and non-TNBC (Table 1).

*Expression levels of circulating miR-155 after treatment*

**Figure 5:**
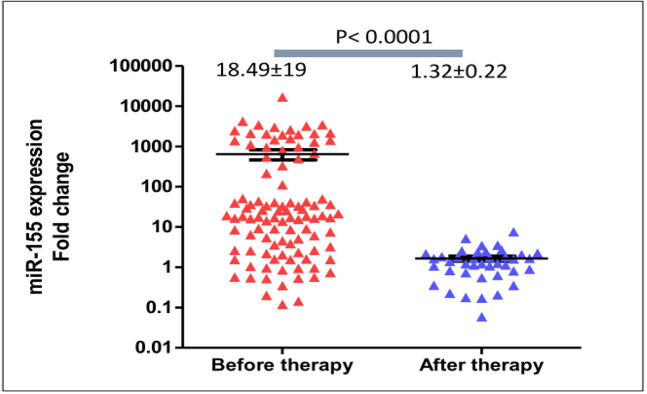
Significant Reduction of Circulating *miR-155* Expression after Surgery and Chemotherapy. In comparison to the baseline, expression of circulating miR-155 was significantly reduced after treatment (medians were 18.49±19 and 1.32±0.22, p<0.0001)

We quantified circulating *miR-155* expression levels in breast cancer patients after completion of surgery and chemotherapy in 40 patients. In comparison to the expression at baseline, circulating *miR-155* expression was significantly lower after treatment (median expression levels were 18.49±218 and 2.14±0.2, respectively; p<0.0001), Figure 5. According to the patient’s age, expression of circulating miR-155 after treatment in patients older than 40 years old were significantly lower than those younger than 40 years-old (means were 1.1±0.14 and 2.35±0.4 respectively, p=0.013) reflecting the larger reduction of circulating *miR-155* in patients older than 40. After treatment, expression levels of circulating *miR-155* were not significantly different between patients with tumor size larger and smaller than 5 cm (means were 1.96±0.43 and 1.43±0.23, p=0.4).

*Upregulation of circulating miR-155 and the correlation with the progression-free survival*

**Figure 6:**
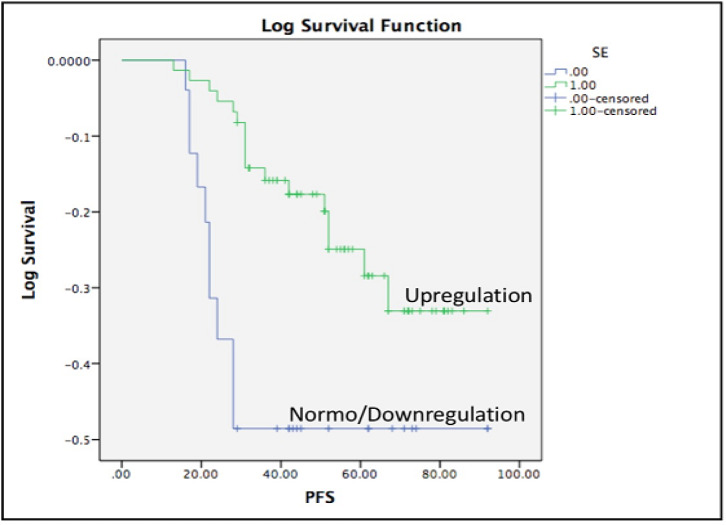
Upregulation of Circulating miR-155 Correlated with Better Progression-Free Survival (PFS). MiR-155 upregulation in breast cancer patients is associated with longer progression-free survival (median PFSs were 77 vs 65 weeks, Mantel-Cox test p=0.038).

We determined upregulation of circulating miR-155 expression at diagnosis as expression above the average expression in healthy women plus 2xSD as explained at the Material and Methods. More than 75% of patients (n=76) showed circulating miR-155 upregulation at diagnosis. Patients with circulating miR-155 upregulation have significant longer progression-free survivals (mean survivals were 77 vs 64 weeks and Mantel-Cox test p=0.038, Figure 6). 

**Table 1:** Clinicopathological Variables of Breast Cancer Patients (N=102) and the Correlation with Circulating miR-155 Expression

Variables /Category	N	miR-155 expression (median±SE)	p-value
Age			
> 40 years-old	84	28.92±20.54	0.0009
≤ 40 years-old	18	4.19±10.5	
Tumor differentiation			
Grade I	9	17.13±10.5	0.67
Grade II	44		
Grade III	49	25.87±25.04	
Tumor size			
≤ 5 cm	45	9.17±6.9	0.03
> 5 cm	57	44.27±2.6	
Stage at diagnosis			
Stage I	3	17.13±10.5	0.52
Stage II	45		
Stage III	51	31.62±23.97	
Stage IV	3		
Subtype			
Luminal	56	25.38±21.4	0.35
Her2	19		
Triple-negative	27	16.09±3.5	

## Discussion

We reported upregulation of circulating *miR-155 *in breast cancer patients and the dynamic changes after completion of surgery and chemotherapy. As developing molecular biomarker in breast cancer has gained interest, the use of circulating microRNA for diagnosis and monitoring therapy is potentially applicable in clinical setting (Schwarzenbach et al., 2011). MicroRNAs are shown to be relatively stable against degradation both in primary tissue samples and body fluids, relatively easy to be detected, and the levels in the tissues and body fluid often reflect the tumor burden and the biological as well as clinical characteristics (Heneghan et al., 2010). Circulating microRNA appears in the plasma through several extracellular transfer modes as a result of high biogenesis and degradation in the malignant cells (Kosaka et al., 2010).* MiR-155 *is frequently upregulated in breast cancer and the higher expression is related with several clinicopathological variables, tumor subtypes, higher tumor grades, and poor survival rates (Chang et al., 2011; Koboldt et al., 2012; Mattiske et al., 2012). Iorio et al. have shown that among 29 deregulated microRNAs in breast cancer primary tissues, only miR-155 and miR-21 are upregulated (Iorio et al., 2005). There are 147 validated miR-155 target genes that have been identified forming interconnecting biological pathways of apoptosis (*CASP3, FADD, FOXO3A*), cell proliferation (*SOCS1*), cell differentiation (*PU.1, MCSFR*), angiogenesis (*HIF*), and epithelial-mesenchymal transition (*TGFβ*) (Mattiske et al., 2012). Although deregulation of miR-155 and the relevant target genes in primary breast cancer tissues have been compiled (Mattiske et al., 2012), only few studies have reported expression of circulating miR-155 and the correlation with clinicopathological variables, effects of treatment, and clinical outcomes in breast cancer. 

We confirmed recent report showing upregulation of plasma miR-155 in breast cancer with a larger number of patient cohort (Khalighfard et al., 2018). In accordance with our results, Lu et al., (2012) reported elevated plasma levels of miR-155 in breast cancer up to 5-fold times than healthy individuals but were higher in patients with small tumor size (2-5 cm) and lymph node infiltration (N1). Liu et al., (2013) also showed upregulation of serum miR-155 in 20 breast cancer patients compared to 10 healthy controls. Using different setting, Gezer et al., (2014) demonstrated significant changes of circulating miR-155 expression upon neoadjuvant treatment in breast cancer, although their findings were contradictory with ours. In accordance with these previous studies, we showed higher circulating *miR-155* expression in breast cancer patients compared to healthy women. A meta-analysis involving 25 studies with total number of 1,896 breast cancer patients and 1,226 healthy controls showed the diagnostic accuracy of circulating miR-155 (Hou et al., 2016). Higher level of plasma miR-155 in early stage breast cancer has also been reported rendering its potential as early detection marker (Gao et al., 2017). 

In response to surgery and chemotherapy, we showed significant decreased circulating miR-155 in breast cancer. Circulating miR-155 levels were also significantly decrease after surgery, chemotherapy, and radiotherapy as reported by a study involving 30 Luminal A breast cancer patients and 10 healthy controls (Khalighfard et al., 2018). Different from this study (Khalighfard et al., 2018), we included larger number of breast cancer patients with different subtypes, although we did not show different expression of circulating miR-155 between Luminal and triple-negative breast cancer subtypes. Further study is required to confirm the potential role of circulating miR-155 as therapeutic monitoring marker in breast cancer including analysis of response into specific chemotherapeutic agent, hormonal therapy, radiotherapy, as well as targeted therapy. With the frequent implementation of neoadjuvant treatment in breast cancer particularly in locally advance stages, the use of circulating miR-155 expression to monitor complete and partial responses to neoadjuvant treatment is also an interesting question in the future. 

Although expression of *miR-155* in primary breast cancer tissue has been associated with the prognosis, limited study has reported the correlation of circulating miR-155 with clinical outcomes (Jang et al., 2017; Lü et al., 2017). In contrast to our previous study using circulating miR-21 (Anwar et al., 2019b), our current study showed that upregulation of circulating miR-155 was associated with better PFS. Although miR-155 is commonly associated as oncomir, our study showed the upregulation with better PFS. In addition, we showed that significant reduction of circulating miR-155 in patients older than 40 years old after surgery and chemotherapy. Potential future research question is whether breast cancer patients with upregulation of circulating miR-155 might response better to the standard treatment. Larger study might be required to explore the potential use of circulating miR-155 as a prognostic marker in breast cancer with particular analysis depending on the subtypes, specific molecular characteristics, and tumor heterogeneity. Although breast cancer in younger women has been associated with more biologically aggressive behavior (Anwar et al., 2019a), we showed higher expression of circulating miR-155 in patients older than 40 years old. Expression of miR-155 interacts with hormonal receptor status and metabolic reprogramming in breast cancer (Bacci et al., 2016; Zeng et al., 2014). However, the direct correlation with age at diagnosis was not previously explained. Mutations at *BRCA1* genes have been associated with younger breast cancer cases and Chang et al., (2011) have shown that particular variations of BRCA1 is able to repress miR-155 expression through modulation of histone acetylation. 

The strengths of this study are relatively larger cohort than previous report (Khalighfard et al., 2018), evaluation of the potential marker after treatment, and thorough analysis in correlation with tumor characteristics and clinical outcome (progression-free survival). Upcoming study evaluating the magnitude decreased circulating* miR-155 *expression after treatment, direct comparison with current biomarker such as CA-15-3, and the correlation with progression-free and overall survival is required. The major constraint of this study is high degree variability of circulating *miR-155* expression that might reflect the diversity of clinical stages, tumor subtypes and biological characteristics of breast cancer patients at diagnosis.
